# Effect of microbubble-assisted gemcitabine delivery with repeated ultrasound exposure in a pancreatic cancer organ-on-a-chip model

**DOI:** 10.1038/s41598-025-30612-2

**Published:** 2025-12-12

**Authors:** Delanyo Kpeglo, Malcolm Haddrick, Margaret A. Knowles, Stephen D. Evans, Sally A. Peyman

**Affiliations:** 1 Molecular and Nanoscale Physics Group, School of Physics and Astronomy, University of Leeds, Leeds, LS2 9JT UK; 2 Institute of Biological Chemistry, Biophysics and Bioengineering, School of Engineering and Physical Sciences, Heriot-Watt University, Edinburgh Campus, Edinburgh, EH14 4AS UK; 3 Leeds Institute of Medical Research at St James’s (LIMR), School of Medicine, University of Leeds, Leeds, LS2 9JT UK; 4 Medicines Discovery Catapult, Block 35, Mereside Alderley Park, Alderley Edge, Manchester, SK10 4TG UK

**Keywords:** Organ-on-a-chip, Microphysiological systems, Tumour mechanics, Tumour microenvironment, Pancreatic ductal adenocarcinoma, Microbubble and ultrasound-assisted therapeutic delivery, Cancer, Engineering, Oncology

## Abstract

The fibrotic stroma of solid tumours poses a physical barrier to drug delivery and effective treatment. Interaction between cancerous epithelial cells and their surrounding stromal partners results in the development of a rigid, collagenous matrix environment with reduced interstitial flow, crucial for drug delivery to cancer cells, particularly in pancreatic ductal adenocarcinoma (PDAC), an aggressive pancreatic cancer with poor prognosis. Therefore, evaluating novel drug delivery mechanisms using appropriate stroma-mimicking 3D culture models is essential. We previously demonstrated, using a 21-day cultured microfluidic PDAC model that mimics the rigid, collagenous stroma, reduced interstitial flow through the tumour model. In this study, we evaluated the use of microbubbles and ultrasound as an alternative method for disrupting our model’s fibrotic stroma to restore interstitial flow and improve gemcitabine delivery and efficacy. Literature shows microbubbles in 2D and 3D static cultures enhance drug delivery and effects by increasing cell membrane permeability through oscillation and bursting under ultrasound (sonoporation). Here, we observed continuous microbubble oscillation and bursting under repeated ultrasound exposure, leading to continuous matrix-microbubble and PDAC cell-microbubble interactions, which improved the gemcitabine effect. This study emphasises the need for disease-specific in vitro models to assess novel drug delivery mechanisms and improve therapeutic outcomes.

**Supplementary Information:**

The online version contains supplementary material available at 10.1038/s41598-025-30612-2.

## Introduction

Pancreatic ductal adenocarcinoma (PDAC) makes up approximately 90% of all pancreatic cancers. It is an aggressive cancer with a 5-year survival rate of < 10%^1–3^. Gemcitabine is the first line of treatment and standard of care for pancreatic cancer patients with locally advanced and metastatic PDAC^4^. It can increase patient survival to approximately 18 months, but due to its side effects, it proves to be an ineffective means of treatment. The dismal prognosis of PDAC stems from an increasingly rigid, fibrotic stroma conferring biophysical forces in the tumour tissue, which defines the disease’s growth and leads to therapeutic resistance^5^.

The rigid fibrotic PDAC stroma arises from the symbiotic relationship between pancreatic cancer cells and stromal components, including pancreatic stellate cells (PSCs), the main fibroblast cells of the pancreas, whose activity is promoted by cytokines and signalling pathways, such as transforming growth factor-β1 (TGF-β1)^2,6–8^. The interaction between the cancer cells and PSCs results in the exacerbated production of extracellular matrix (ECM) proteins, of which collagen type I is the most abundant in the PDAC tumour tissue^9–11^. The accumulation of ECM proteins and increasing tumour mass leads to tissue stiffness, high interstitial pressure, and reduced interstitial flow, which is a critical barrier for drug delivery to the cancer cells^12–14^.The extremely poor survival rate of PDAC, as a result of the fibrotic tumour microenvironment, warrants an urgent need for novel therapeutics to improve patient outcomes. However, relevant in vitro models that accurately mimic these biophysical barriers in the PDAC tumour, such as stroma rigidity and reduced interstitial flow, are also needed to develop and test new therapeutics.

 In vitro two-dimensional (2D) and three-dimensional (3D) culture models, as well as mouse models, have helped improve the modelling of tumours for drug testing^15–19^. However, many of these studies fail to recapitulate the biophysical characteristics of the PDAC tumour tissue, which are critical in the disease’s ability to resist therapy. The models presented either do not embody the 3D tumour microenvironment complexities, such as cell–matrix interactions (with 2D culture models), are often in static fluidic conditions and are mechanically immature with a 3—7 day culture (2D and 3D culture models), and with interspecies differences (mouse models) results in drug failure during clinical trials^15,20–22^.

Microfluidics and organ-on-a-chip technology enable precise control of fluid flow within defined geometries, creating microphysiological systems that are more closely aligned with in vivo tumour tissue^23–26^. Microfluidic-based cell culture models of PDAC, with cells seeded in a collagen-rich matrix for a culture period of approximately 3–10 days, have demonstrated how the PDAC stroma can influence drug delivery to the cancer cells and their effects^27–30^. However, the cultures presented are either epithelial cells only, murine-derived, or based in a well-plate format, which neglects the dynamic hydraulic environment of tissues. Therefore, they do not represent the PDAC tumour microenvironment and are not mechanically mature, demonstrating the fibrous stroma for drug studies.

We have previously shown that the in vivo PDAC tissue stroma and mechanical stiffness can be recapitulated in vitro using a microfluidic platform with a 21-day culture of PDAC cancer cells, PSCs, and TGF-β1 supplement. While our model does not fully recapitulate the complexity of the PDAC tissue with the absence of endothelial and immune cells, we focused on developing a foundational model centred around the dense, rigid PDAC fibrotic stroma and demonstrated an accumulation of collagen in the culture environment, resulting in reduced interstitial flow and resistance to gemcitabine effects^31,32^. The use of microfluidics as a culture platform permitted the perfusion of the culture, similar to interstitial flow, to adequately model the hydraulic environment of the PDAC stroma (*i.e.,* reduced interstitial) and investigate its role in drug delivery and how to improve gemcitabine efficacy. We demonstrated that disrupting the fibrotic stroma with the anti-hypertensive drug losartan, which has matrix-depleting properties, enabled the reversal of the reduction in interstitial flow and enhanced the effect of gemcitabine against PDAC cells^32^. However, pretreatment with losartan is not targeted to the tumour only, and the investigation of new routes to deplete PDAC’s rigid stroma needs to be more focused on the tumour to limit damage to healthy tissues.

Microbubbles are approved contrast agents for diagnostic ultrasound (US) that have recently been given attention as potential new routes for delivering drugs in the body. Microbubbles are phospholipid or protein-shelled bubbles of about 1—10 µm in diameter with an inert gas core, such as perfluorocarbon or sulphur hexafluoride.^33,34^ The use of microbubbles with US has also been shown to increase cellular drug uptake due to the microbubbles oscillating under US exposure and the effect this has on the membrane permeability of nearby cells. This phenomenon is known as sonoporation and can be described as a US ‘triggered’ therapy^35,36^. In addition, the US transducer can be focused around the tumour area, so this therapeutic effect is minimised in healthy tissues. US and microbubble-assisted gemcitabine delivery to PDAC cancer cells has been used in vitro on 2D cultures and in vivo with orthotopic models and shows how microbubbles and US promote the effective transport of gemcitabine to cells, decreasing cell viability and tumour volume.^37–39^ Clinically, US and microbubble have been tested in patients with inoperable pancreatic cancers and have resulted in a median survival of 17.6 months compared to control groups (~ 8.9 months) and literature values (9.3 months)^40,41^. Moreover, microbubbles can be modified to attach drug molecules, nanoparticle carriers, or drug-loaded liposomes to the phospholipid shell, thereby increasing drug delivery and availability to cancer cells while causing less harm to healthy cells and tissues^42–45^.

To better assess these new therapeutics against PDAC cancer cells, accurate in vitro models that recapitulate the stiff PDAC stroma and its biophysical hallmarks are of vital importance. Only by selecting effective therapeutics against accurate models of the disease can we hope to improve patient outcomes. Here, using our microfluidic PDAC model cultured for 21 days to achieve mechanical maturity, we investigated the effect of microbubbles with repeated US exposure on restoring interstitial flow and enhancing gemcitabine efficacy against the PDAC cancer cells.

### Experimental section

#### Cell lines and culture

Pancreatic ductal adenocarcinoma cells, PANC-1 (ECACC 87,092,802)^46^, were maintained in Dulbecco’s Modified Eagle Medium (DMEM; ThermoFisher Scientific) supplemented with 10% FBS (Sigma Aldrich), 1% Penicillin Streptomycin (P/S; Sigma-Aldrich), and 1% GlutaMAX (Thermo Fisher Scientific). Human pancreatic stellate cells (PSCs) were maintained in stellate cell medium supplemented with 10% FBS, 1% stellate cell growth supplement, and 1% P/S in culture flasks coated with poly-l-lysine (10 mg mL^−1^). The PSCs and respective culturing reagents were sourced from ScienCell™ Research Laboratories, supplied by Caltag Medsystems Ltd. The PANC-1 and PSCs were cultured at 37 °C with 5% CO_2_ under humidified conditions (95—99%). The cells were passaged using TrypLE Express Enzyme (1 ×) without phenol red (ThermoFisher Scientific) and used once confluence was achieved at  ≥70%. PSCs were used up to passage 6.

#### Microfluidic device design and fabrication

A co-culture model of PDAC was grown in a 5-channel microfluidic device, previously described^32^. Briefly, topographically, the 5-channel microfluidic device comprised a 1 mm × 6 mm (w × L) culture chamber, two gel containing channels measuring 275 µm in width, and two media channels measuring 100 µm in width, positioned adjacent to the gel channels. At the channel boundaries, micropillars with a 5 µm interspace were incorporated to help confine cells and gel within their respective channels and to ensure stable diffusion of medium with nutrients into the culture chamber for growing our PDAC model and with microbubbles and gemcitabine when treating our model. Supplementary Fig. 1 shows the CAD schematic of the device.

A master mould of the 5-channel microfluidic device was designed using Autodesk’s AutoCAD® software and fabricated in-house using photolithography with SU-8 2075 negative photoresist (Microchem, Newton, MA) and PDMS soft lithography with base PDMS and cross-linking agent (Sylgard™ 184 Silicon Elastomer Kit). The PDMS moulds of the devices were bonded onto PDMS-coated (50 µm thick) glass slides using O_2_ plasma and autoclaved before cell culture.

#### Microfluidic PDAC culture

As previously described^32^, 1 × 10^6^ cells mL^−1^ of PDAC cells (co-culture of PANC-1 and PSC cells; 1: 3 seeding ratio) was mixed with 6—9 mg mL^−1^ of basement membrane extract (BME) gel, pipetted into the culture chamber of the 5-channel microfluidic device and incubated for 30 min under humidified conditions at 37 °C with 5% CO_2_. After incubation, by pipetting, the gel containing channels were filled with 9—12 mg mL^−1^ of BME gel. The device was then placed into the incubator to polymerise the gel for another 30 min. Culture hydrostatic reservoirs^47^ were then inserted into the respective inlets and outlets of the culture medium channels and filled with DMEM/10% FBS culture^29^ medium supplemented with TGF-β1 supplement (10 ng mL^−1^) for a 21-day culture under humidified conditions at 37 °C with 5% CO_2_.

#### Gemcitabine-only assessment

Gemcitabine (Sigma Aldrich) was solubilised with DMSO (Sigma Aldrich) to prepare a 5 mg mL^−1^ stock solution and stored at − 20 °C until use. The stock solution was diluted with DMEM/10% FBS solution to a concentration of 31.25 µM and used to treat the 21-day microfluidic PDAC cultures for 72 h. On-chip culture viability was assessed by quantifying the ATP content with CellTiter-Glo® 3D cell viability assay (Promega), as previously described^32^. Briefly, according to the manufacturer’s instructions, an equal volume of the CellTitre-Glo reagent was added to the culture medium present in the hydrostatic reservoirs with the on-chip model for incubation. After incubation, the effluent and the whole on-chip culture model were removed by pipetting into opaque plates for luminescence reading with a microplate reader (SpectraMAX M2, Molecular Devices). The viability was normalised to positive and negative controls to determine the effect of gemcitabine on the microfluidic PDAC culture.

#### Microbubble preparation

Microbubbles were prepared by mixing DPPC, DSPE-PEG2000, and DOPE-ATTO 488 in a molar ratio of 95: 4.9: 0.1% for a final phospholipid concentration of 2 mg mL^−1^. The DPPC and DSPE-PEG2000 lipids were purchased from Avanti Polar Lipids (USA), and the DOPE-ATTO 488 lipids, used to fluorescently label the microbubbles, were purchased from ATTO-TEC (Germany). The lipid solution was dried under a steady stream of nitrogen for up to 1 h and left under vacuum overnight to remove the chloroform: methanol storage solution in which the lipids were dissolved. After drying, the lipid solution was rehydrated by resuspending in sodium chloride (NaCl) solution containing 1% glycerol (vol/vol), then sonicated in a water bath to help incorporate DOPE-ATTO 488 into the phospholipid shell, without requiring chemical conjugation, given that ATTO488 is hydrophilic. The hydrated lipid solution was then combined with perfluorobutane (C_4_F_10_) gas in a multiplexed microfluidic device^48^ for microbubble production. After production, the microbubbles were imaged with an upright epi-fluorescence microscope (E600, Nikon, Tokyo, Japan), and the size and concentration were analysed using a MATLAB-based Microbubble Population Analysis code program^49^.

#### Gemcitabine, microbubbles, and ultrasound assessments with the microfluidic PDAC culture

PDMS mould of the 5-channel device used for the 21-day on-chip culture for US exposure was fabricated to a 1 mm thickness to reduce US attenuation. However, for optimal culturing with the culture hydrostatic reservoirs, additional PDMS of 4—5 mm thickness was bonded onto the inlet and outlet media regions of the device. In our experimental setup (Supplementary Fig. 2), the ultrasound path through the PDMS of our microdevice is short, with a PDMS thickness of only 1 mm and approximately 6 mm of gel pad on top of the culture area for ultrasound coupling. Acoustic attenuation should therefore be minimal at low mechanical indices of propagation, consistent with previous reports^50,51^. The 21-day microfluidic PDAC cultures were treated with microbubbles mixed in a 1:10 ratio with 31.25 μM gemcitabine in DMEM/10% FBS culture medium solution. To minimise mechanical and thermal effects, avoiding cellular damage, while promoting controlled stable and inertial cavitation dynamics^52,53^, US was applied once, or every 5 min up to 30 min, using an unfocused transducer with a central frequency of 2.25 MHz (V323-SM, Olympus, Tokyo, Japan) and an element diameter of 6.35 mm, which covers the width of our microfluidic culture model region (channel width is 6 mm; see Supplementary Figs. 1 and 2) for a total duration of 5 s at a mechanical index of 0.6, corresponding to a peak negative pressure of approximately 900 kPa, with a pulse repetition frequency of 1 kHz, and a duty cycle of 1%. As described previously^47^, the US pulses were controlled by a signal generator (TG5011, Agilent Technologies, UK) driven by a + 53 dB amplifier (A150, Electronics & Innovation, Rochester, USA) to generate the US signal to the microfluidic PDAC cultures. The transducer was coupled to the devices with the PDAC culture with a gel pad (AquaFlex, Parker Laboratories, US) of approximately 6 mm and US transmission gel (Anagel®, AnaWiz Ltd). High concentrations of microbubbles can result in acoustic shadowing or attenuate ultrasound pressure^54,55^. However, we believe that significant shadowing was unlikely in this ultrasound setup due to the short ultrasound path length and the confined geometry limiting attenuation compared to in vivo tissues. The cultures were imaged after US application using a Leica-TCS-SP8 confocal laser scanning microscope with a 10 × objective and a pinhole of 1.00 AU with the respective excitation and emission wavelength for the ATTO 488 lipid in the phospholipid shell of the microbubbles. Acquired images were analysed with Image J. Culture viability after incubation with the different treatments was assessed by quantifying the ATP content as briefly described above (in *Gemcitabine-only assessment*). The viability was normalised to positive and negative controls to determine the effect of gemcitabine on the microfluidic PDAC culture.

#### Gemcitabine, microbubbles and ultrasound assessments with 2D PDAC cultures

For comparison, PANC-1 and PSC cells, in a 1: 3 ratio at 5—7 × 10^5^ cells mL^-1^, were seeded into 96-well or ibidi μSlide (VI 0.4, iBidi, Germany) devices for 2D culture with TGF-β1 supplement (10 ng mL^−1^) until ≥ 70% confluence was achieved. The 2D cultures were treated with gemcitabine-only or microbubbles in gemcitabine solution. US was applied using the 2.25 MHz transducer at a mechanical index of 0.6, a pulse repetition frequency of 1 kHz, and a duty cycle of 1% for 5 s. The cultures were incubated for 72 h prior to ATP viability assessment with CellTiter-Glo® 2D reagent (Promega). According to the manufacturer’s instructions, a volume of the reagent equal to the volume of culture media present in the 96-well and ibidi μSlide devices was added for incubation. After incubation, the contents of the 96-well plate and ibidi μSlide devices were removed by pipetting into opaque plates for luminescence reading with a microplate spectrophotometer reader (SpectraMAX M2, Molecular Devices).

### Statistical analyses

Data were expressed as mean ± standard error (SE) for biological and experimental replicates, and statistical significance was assessed using OriginPro software. Normality and equal of variance were tested, and once normality and equality of variance assumptions were satisfied, significance was measured with one-way ANOVA, followed by Tukey’s multiple comparisons test. *p* ≤ 0.05 was considered statistically significant.

## Results and discussion

Despite advancements in understanding the genetic hallmarks for therapeutic targets to improve treatment outcomes for PDAC patients, recent studies have shown that the tumour microenvironment is central to drug resistance^2,31,56,57^. The crosstalk between PDAC cancer cells and the tumour microenvironment, predominantly involving fibroblasts or cancer-associated fibroblasts (CAFs), results in a cellular environment rich in CAFs, growth factors, and abundant ECM proteins for a rigid, fibrotic environment, which remains one of the primary reasons for poor therapeutic efficacy^2,57–60^ However, there is a lack of in vitro models that accurately mimic the rigid, fibrotic stroma of the disease, hindering the evaluation of novel therapeutics to improve patient outcomes.

We have previously demonstrated the ability to mimic the mechanics of the PDAC tumour microenvironment off-chip with 3D spheroid cultures and on-chip with a 5-channel microfluidic device^31,32^. Due to the advantage microfluidics provides in developing appropriate 3D culture environments mirroring the in vivo tissue over 2D and 3D static well-plate formats, we used our 5-channel microfluidic device to grow PDAC cells and have shown with losartan treatment (an angiotensin II receptor blocker with matrix depleting properties) that disrupts the rigid stroma environment, there was increased interstitial flow for improved gemcitabine effect^32^. However, losartan is not targeted solely at the tumour, whereas other new delivery mechanisms, such as MBs, can be targeted specifically at the tumour and limit damage to healthy cells and tissues. Here, with the use of microbubbles and US to improve drug delivery and effect, we further highlight measures to increase interstitial drug penetration, which is crucial to targeting the PDAC cancer cells.

### The microfluidic PDAC culture and the effect of gemcitabine-only treatment

**Fig. 1:**
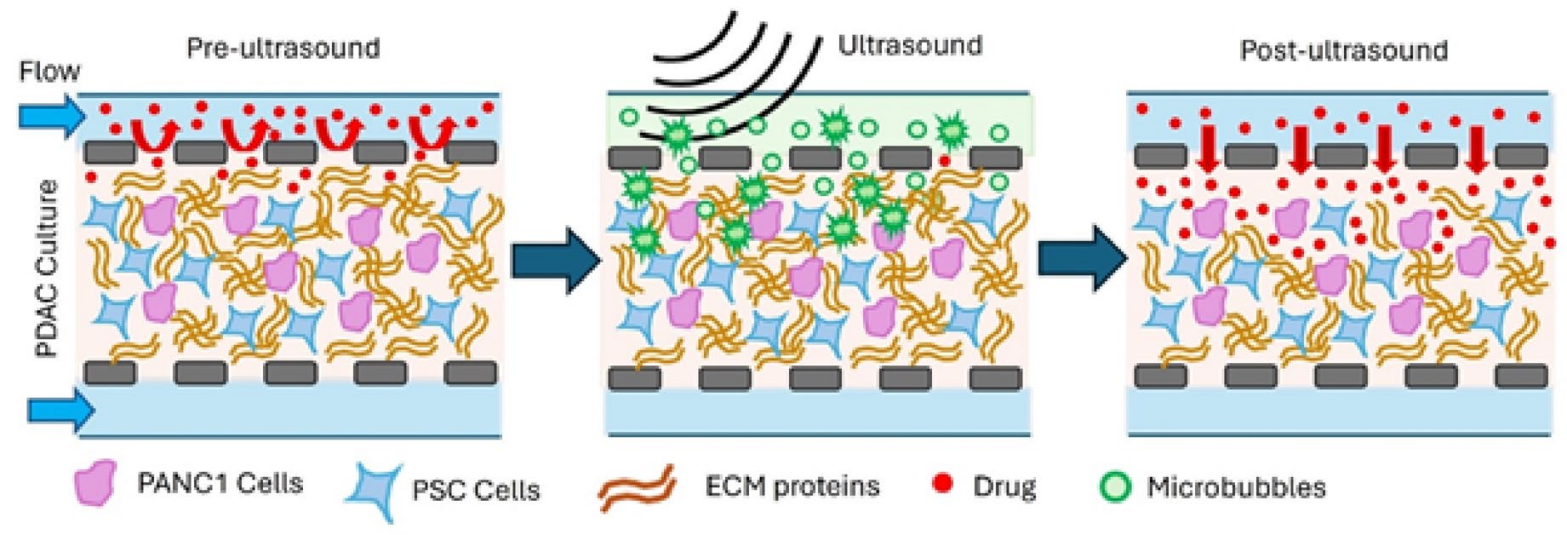
Conceptual schematic of the microbubble and US treatment of the on-chip PDAC culture showing the application of US bursts microbubbles, disrupting the matrix and increasing the interstitial flow of drugs into the culture. Not to scale.

Figure 1 shows a schematic of the on-chip PDAC culture treatment with microbubbles and US, demonstrating that following the disruption of the matrix by bursting the bubbles, drug penetration into the PDAC culture increases, thereby improving efficacy. Supplementary Fig.1 shows the CAD schematic of the 5-channel microfluidic device used for growing the PDAC culture model. The PDAC cells, consisting of PANC-1 cancer cells and pancreatic fibroblast cells (PSCs) were seeded into the 5-channel microfluidic device for a 21-day culture with TGF-β1 supplement. This was to ensure the PDAC culture reflected the mechanical environment (a mechanically stiff, collagenous environment with reduced interstitial flow) of the PDAC tumour tissue^2,32,57,61^. The 5-channel device consisted of two additional gel containing channels on either side of the main culture chamber to help support cell growth over 21 days. The gel containing channels ensured the PDAC cells in the culture chamber did not grow into the culture medium channels and, therefore, impeded the flow of nutrients into the culture chamber with the PDAC cells. The use of the hydrostatic reservoirs^47^ eliminates issues with the use of cumbersome syringe pumps and tubing in the cell culture incubator and permits the culturing of the PDAC cells on-chip under flow, which is representative of the physical environment of cells in tissues^62^.

**Fig. 2:**
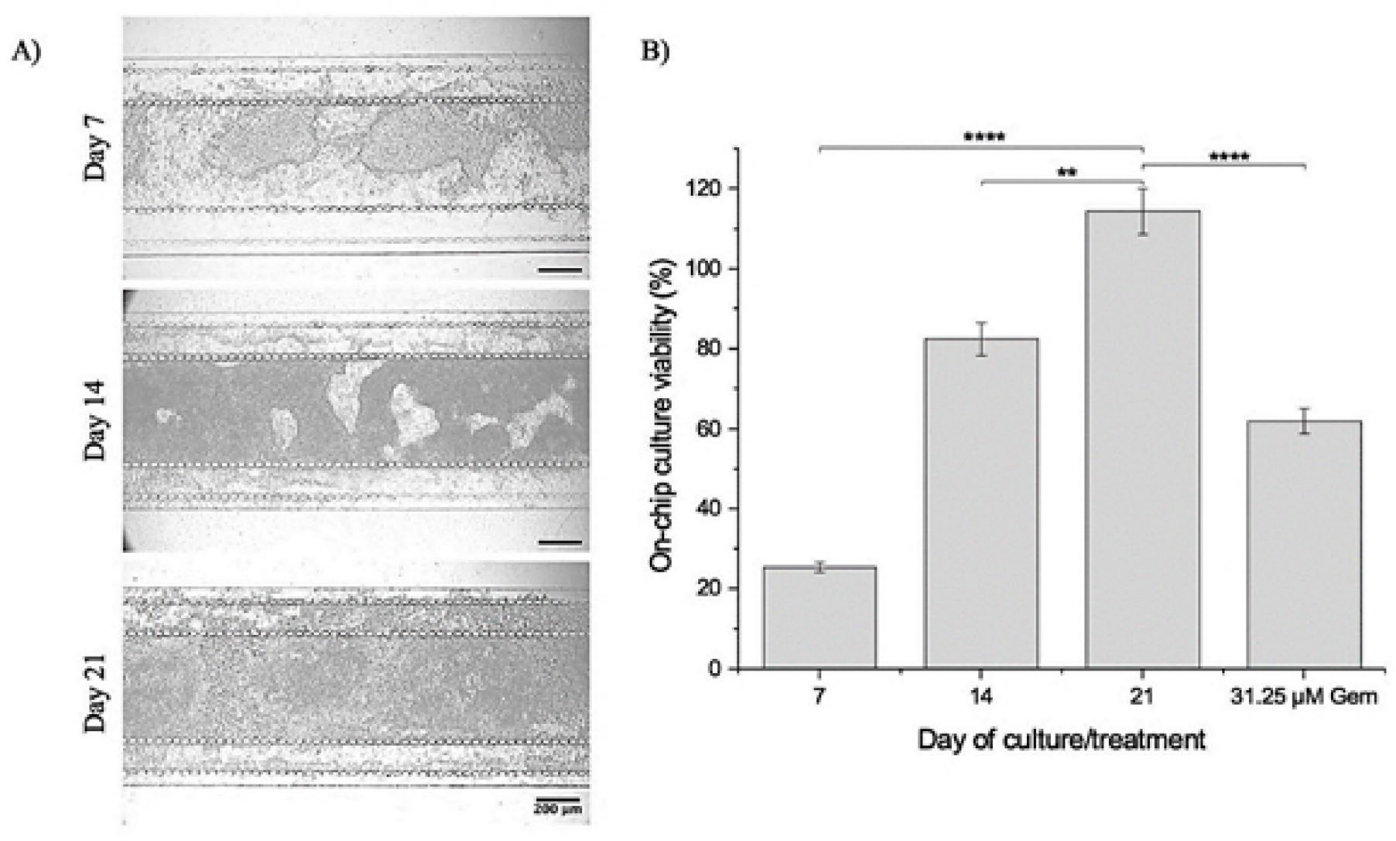
(**A**) Bright-field images of the 21-day microfluidic PDAC culture model. Scale bar, 200 μm. (**B**) ATP viability assessment of the culture on days 7, 14, and 21 of culture without treatment after a 72-h incubation, where *n* = 18 devices per day of culture generated from three separate seeding settings. With 31.25 µM of gemcitabine at day 21, *n* = 12 on-chip cultures generated from three separate seeding settings. ****p < 0.001, and **p < 0.01, one-way ANOVA followed by Tukey’s multiple comparison test.

Figure 2A shows a representative bright-field image of the PDAC cultures from day 7 to day 21, showing growth inside the culture chamber. Figure 2B shows the ATP viability assessment of the PDAC cells grown in the device during the 21-day culture period and following treatment with 31.25 µM of gemcitabine for 72 h. The concentration of 31.25 µM gemcitabine was found to be an optimum concentration for assessing improvements in drug delivery from previous assessments by demonstrating cell kill, but with enough cell viability remaining that improvements to the drug delivery mechanism could be assessed, such as delivery with microbubbles and US^31,32^. Figure 2B shows there was increased culture viability, indicating the cultures were growing and viable in the 5-channel device over the 21-day culture period without treatment. Accumulation of collagen over this period reduces the size of the porous matrix structure and, therefore, results in reduced interstitial flow^12,13,63^. Interstitial flow is essential for delivering nutrients and removing waste from cells. A decrease in the interstitial flow also leads to the ineffective delivery of chemotherapeutics to cells, resulting in poor drug effects^12^. With gemcitabine-only treatment at 31.25 µM, the percentage viability for the 21-day microfluidic culture decreased to 62%. As shown in our previous assessments, when the 7-day microfluidic PDAC cultures, which exhibit a soft ECM, were treated with 31.25 µM gemcitabine, culture viability decreased to only 9.8%. Compared to the 21-day culture, this was approximately an 84% decrease in culture viability, demonstrating the impact the mechanical rigidity of a 21-day culture had on reducing the effectiveness of gemcitabine^32^. On day 7 of culture, with the cells degrading and remodelling their culture matrix to support their growth^31^, there would be high interstitial flow through the culture, delivering gemcitabine to the cells compared to the culture on day 21, where there was a rigid matrix environment with reduced interstitial flow demonstrating the importance of the rigid 21-day culture in mimicking drug resistance in PDAC^12,32,64^.

### Effect of microbubbles and ultrasound exposure on the microfluidic PDAC culture

Known as sonoporation, it is well documented that as microbubbles oscillate and burst under the influence of US, they produce tiny, powerful shock waves that can penetrate localised cell membranes and increase cellular drug uptake^54,65,66^. Furthermore, due to the focused nature of the US, the therapy is targeted to the region of interest, such as a tumour, reducing the effect in distant healthy tissue. Typically, the cavitation of microbubbles occurs within the vasculature or near vascular endothelium. However, the effects of cavitation, such as increased permeability and localised disruption, can extend beyond the vessel wall and influence the surrounding tissue parenchyma^67,68^. Given the nature of these cavitation or shockwaves, it was therefore hypothesised that the same shockwaves produced by microbubble oscillation and bursting under US could potentially disrupt the matrix network of our tumour model, as the cavitation-induced effects of the microbubbles extends from the culture medium channels, across the 5 μm interspaced micropillars, into and across the gel containing and culture channel with our model culture, to help restore interstitial flow and increase cell membrane permeability for an improvement in gemcitabine uptake and effect^37,54,69,70^. First, the ability of microbubbles to penetrate through the PDAC culture, their effect on the culture viability without gemcitabine, and the effect of US exposure with and without microbubbles on the culture viability were assessed as controls.

**Fig. 3:**
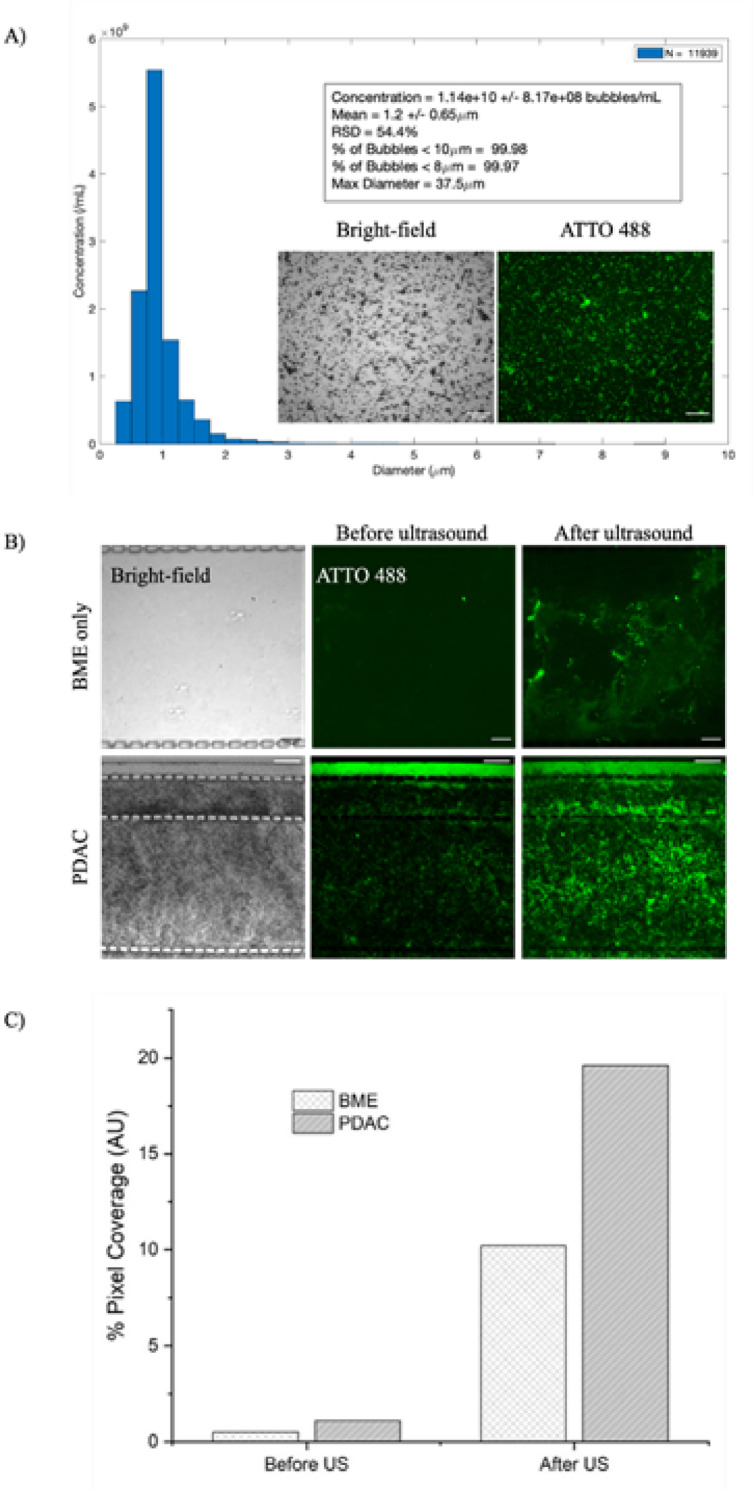
(**A**) Size histogram and concentration of microbubbles. The insert shows the ATTO 488 signal from the lipid shell. (**B**) Brightfield and fluorescence confocal images of BME gel only and PDAC cultures with microbubbles, both before and after US exposure. The scale bar is 100 µm for the BME gel and 200 µm for the PDAC. (**C**) Percentage coverage of pixel intensity before and after US exposure for BME only and PDAC cultures, analysed using ImageJ.

DPPC: DSPE-PEG2000: ATTO 488 microbubbles were produced on a multiplexed microfluidic device^48^. The device provides an atomisation effect with flow-focusing nozzles for the production of microbubbles in the micro-spray regime at a concentration of ≥ 1 × 10^8^ microbubbles per mL and with a mean diameter of < 2 µm^48,71^. Figure 3A shows a histogram of microbubble size distribution with a mean diameter of 1.2 ± 0.65 µm and a concentration of 1.4 × 10^10^ ± 8.17 × 10^8^ bubbles per ml. The insert in Fig. 3A shows microbubbles with ATTO 488 in the shell, displaying a green fluorescence ring around the microbubbles. For on-chip assessments, the microbubbles were diluted with DMEM/10% FBS culture medium in a 1:10 ratio and perfused into the devices using hydrostatic reservoirs for about 2 h. As a control, the flow of microbubbles through the chamber was also assessed with BME gel only and without cells. Imaging was performed using confocal microscopy with a 10 × objective and a pinhole of 1.00 AU, with the respective excitation and emission wavelengths for the ATTO 488 lipids. Figure 3B shows brightfield and fluorescence images of areas of the culture channel containing BME only or the PDAC culture before and after the application of US. Green signal from the ATTO 488 lipids in the microbubble shell can be seen in the fluorescence images. Figure 3C shows data from analysing the percentage area of bright pixel coverage in the fluorescence images of the PDAC culture before and after US application, with an increase from 0.5 and 1.1% to 10.2 and 19.6% for BME only and PDAC model, respectively. Supplementary videos 1 and 2 show the flow of the microbubbles passing between the pillars into the culture chamber of the device with the PDAC model and BME gel only, respectively.

When microbubbles were allowed to flow through the media channels of the microfluidic device, a small number could be observed permeating either the BME gel or the PDAC culture. Our previous studies indicated that a 21-day culture of PDAC within this device resulted in reduced interstitial flow due to the dense matrix formed by the culture^32^. This supports the observation that only a small number of microbubbles were seen penetrating the culture. Following the application of US, there was an increase in the fluorescence signal in both the BME and the PDAC chambers, suggesting that the destruction of the microbubbles not only released their fluorescent lipid shell into the surrounding matrix, but also indicated that there was a further increase in the number of microbubbles entering the chambers, indicating a disruption of the gel or matrix, resulting in an increase in interstitial flow through the chamber. For the BME only gel, this was an increase from 0.5% bright pixel area coverage to 10.2%, and for the PDAC culture, from 1.1 to 19.6%. The difference in permeability and disruption between the BME only and PDAC cultures is not entirely understood but could be down to factors such as heterogeneity in the network porosity from the complex matrix composition the PDAC model has, with *e.g.,* collagenous proteins, integrins, proteoglycans, glycoproteins, and proteases^72^, in comparison to the BME gel, which has been previously shown to be mechanically weak^31^. As a control, it is important to understand how energy from the US may affect the PDAC culture’s permeability and viability. Supplementary Fig. 3 shows brightfield images of the PDAC culture and BME only gel before and after exposure to US without the presence of microbubbles. The images show no observable disruption of the gel or the PDAC culture with US only.

### The effect of gemcitabine, microbubbles, and single ultrasound exposure on the microfluidic PDAC culture

Studies with microbubbles and US exposure with gemcitabine treatment, in vitro and in vivo, have demonstrated increased gemcitabine uptake and efficacy^37,38,73^. However, 2D cultured cell lines and mice models have been used for these assessments, and the effect of gemcitabine, microbubbles, and US together have not been investigated on mechanically stiff 3D PDAC cultures or with a microfluidic PDAC culture, where the in vivo-like microphysiological environment is better recapitulated. DPPC: DSPE-PEG2000: microbubbles with DMEM/10%/ 31. 25 µM gemcitabine solution in a 1:10 ratio was introduced into the culture chamber of the 21-day microfluidic PDAC culture model, and US was applied for a total duration of 5 s. Culture viability was assessed after 72 h. As controls, the cultures were treated with gemcitabine only (Gem), microbubble only (MBs), ultrasound only (US), gemcitabine and microbubbles together (Gem + MBs), and gemcitabine and US together (Gem + US) to compare the effects of gemcitabine, microbubbles, and US together (Gem + MBs + US).

**Fig. 4:**
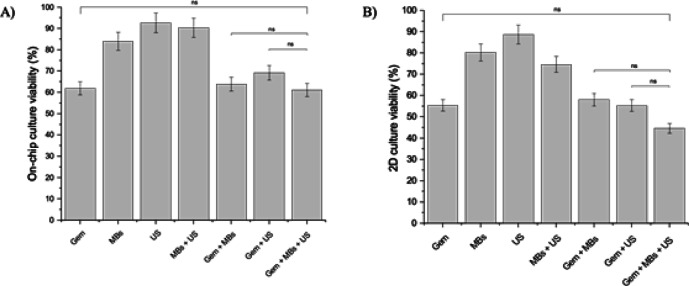
ATP viability assessment of the PDAC cultures grown on-chip with the 5-channel microfluidic device and in 2D. (**A**) Viability of the microfluidic PDAC cultures treated with 31.25 µM gemcitabine, microbubbles, and ultrasound. *N* = 6—8 microfluidic PDAC cultures for the microbubble-only (MBs), ultrasound only (US) and microbubbles and ultrasound together (MBs + US) treatments, *n* = 12 microfluidic PDAC cultures for gemcitabine only (Gem) treatment, *n* = 7 microfluidic PDAC cultures for the gemcitabine and microbubbles together (Gem + MBs) and gemcitabine and ultrasound together (Gem + US) treatments, and *n* = 9 microfluidic PDAC cultures for the gemcitabine, microbubbles, and ultrasound together (Gem + MBs + US) treatment, all generated from three separate seeding settings. B) Viability of the PDAC cells grown in a 96-well plate and ibidi µSlide devices and treated with 31. 25 µM gemcitabine, microbubbles, and ultrasound. *N* = 6 wells of a 96-well plate and 6 ibidi µSlide channels for each of the treatment conditions from three separate seeding settings. *Ns*, p > 0.5, one-way ANOVA followed by Tukey’s multiple comparison test.

Figure 4 shows the ATP viability assessment of the PDAC culture, grown in the microfluidic device and 2D in a 96-well plate and ibidi μSlide devices for comparison after treatment with gemcitabine, microbubbles, and US together. For the control groups, Fig. 4A shows that the percentage viability of the PDAC model with MBs, US, and microbubbles with US (MBs + US) was 84%, 93%, and 90%, respectively. This indicates that each control group had no significant detrimental effect on the PDAC culture viability, and a reduction in culture viability could be attributed to therapeutic treatment. Figure 4B shows viability assays for the 2D cultures, with the control groups showing similar viability; the MBs and US treatments were not detrimental to the culture viability. For assays involving the chemotherapeutic gemcitabine, Fig. 4A shows the percentage viabilities for the 21-day microfluidic PDAC culture model with Gem, Gem + MBs, Gem + US and Gem + MBs + US with a reduction in PDAC viability compared to controls of approximately 52%, 50%, 45%, and 53%, respectively. As expected, the presence of gemcitabine showed a lower culture viability compared to the control groups. However, there was no significant difference between the percentage viabilities, particularly between the Gem and Gem + MBs + US treatments. This indicates that, although the presence of microbubbles in the chamber was observed, they did not significantly contribute to any additional therapeutic effect compared to gemcitabine alone. The reason for this is that the level of matrix disruption caused by a single application of US to the PDAC culture was likely insufficient to significantly increase interstitial flow and provide an observable improvement in the gemcitabine effect. For 2D PDAC cultures in a 96-well plate or ibidi µ-Slide devices,

Figure 4B shows the percentage viability assessment, with no significant difference in the percentage viabilities between the Gem and Gem + MBs + US treatments, resulting in a 46% and 56% reduction in viability, respectively. However, compared to the microfluidic culture model, there was a further decrease in culture viability by approximately 11% and 26% for the Gem and Gem + MBs + US treatment, respectively. This highlighted that the microfluidic PDAC culture mechanics influenced the resistance of the culture to Gem and Gem + MBs + US treatments, an effect also mirrored in vivo. 2D cultured cells lack the 3D complexities seen in the in vivo tissue, as exemplified by our microfluidic PDAC culture.

### Effect of gemcitabine, microbubbles, and repeated ultrasound exposure on the microfluidic PDAC culture

**Fig. 5:**
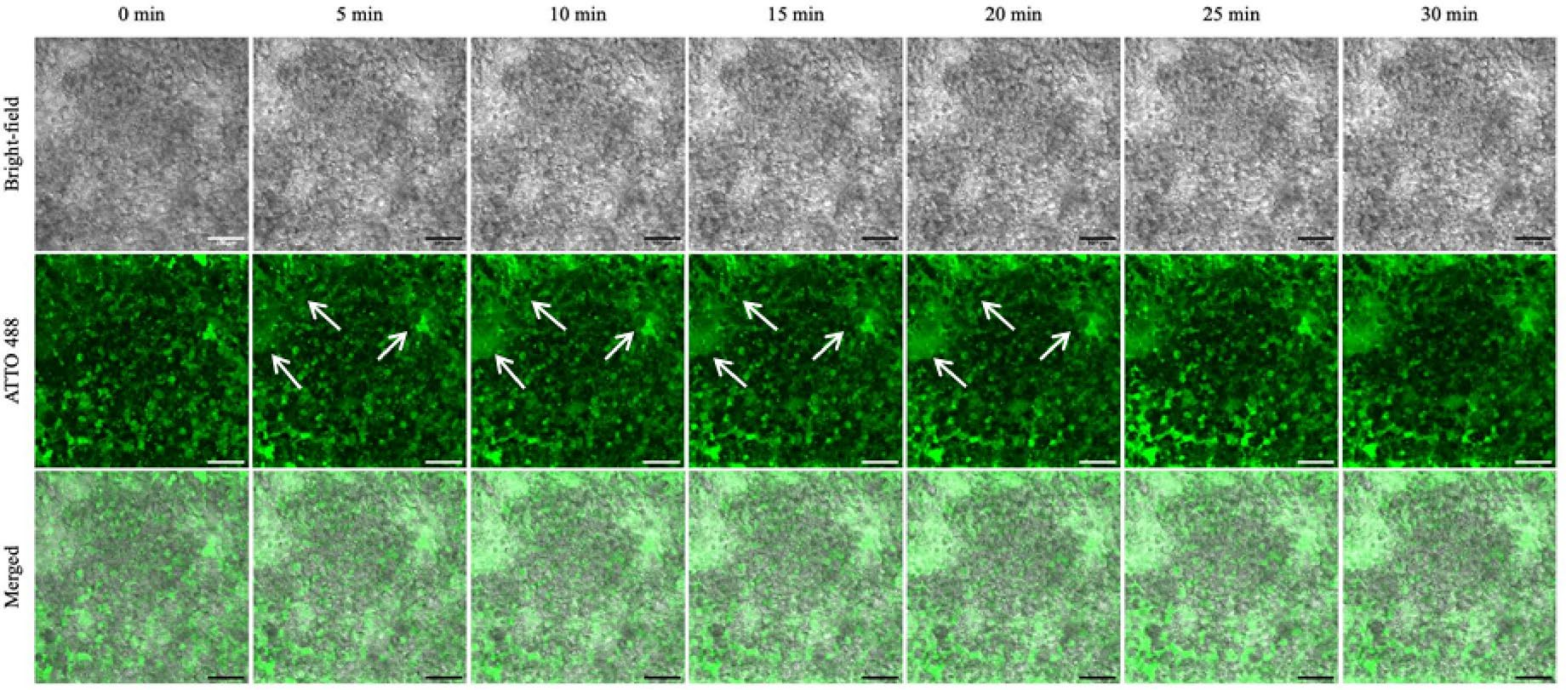
Confocal images of the ATTO 488 fluorescence intensity in the 21-day microfluidic PDAC culture with repeated ultrasound exposure. Gemcitabine and microbubble solution was perfused on-chip with the PDAC culture for 2 h to allow bubbles in the media channel to penetrate the PDAC culture. With microbubbles in the culture chamber, ultrasound was applied every 5 min for 30 min. White arrows indicate the changes in the ATTO 488 signal from the microbubble shell during the 30-min US exposure. Scale bar, 100 µm.

**Fig. 6:**
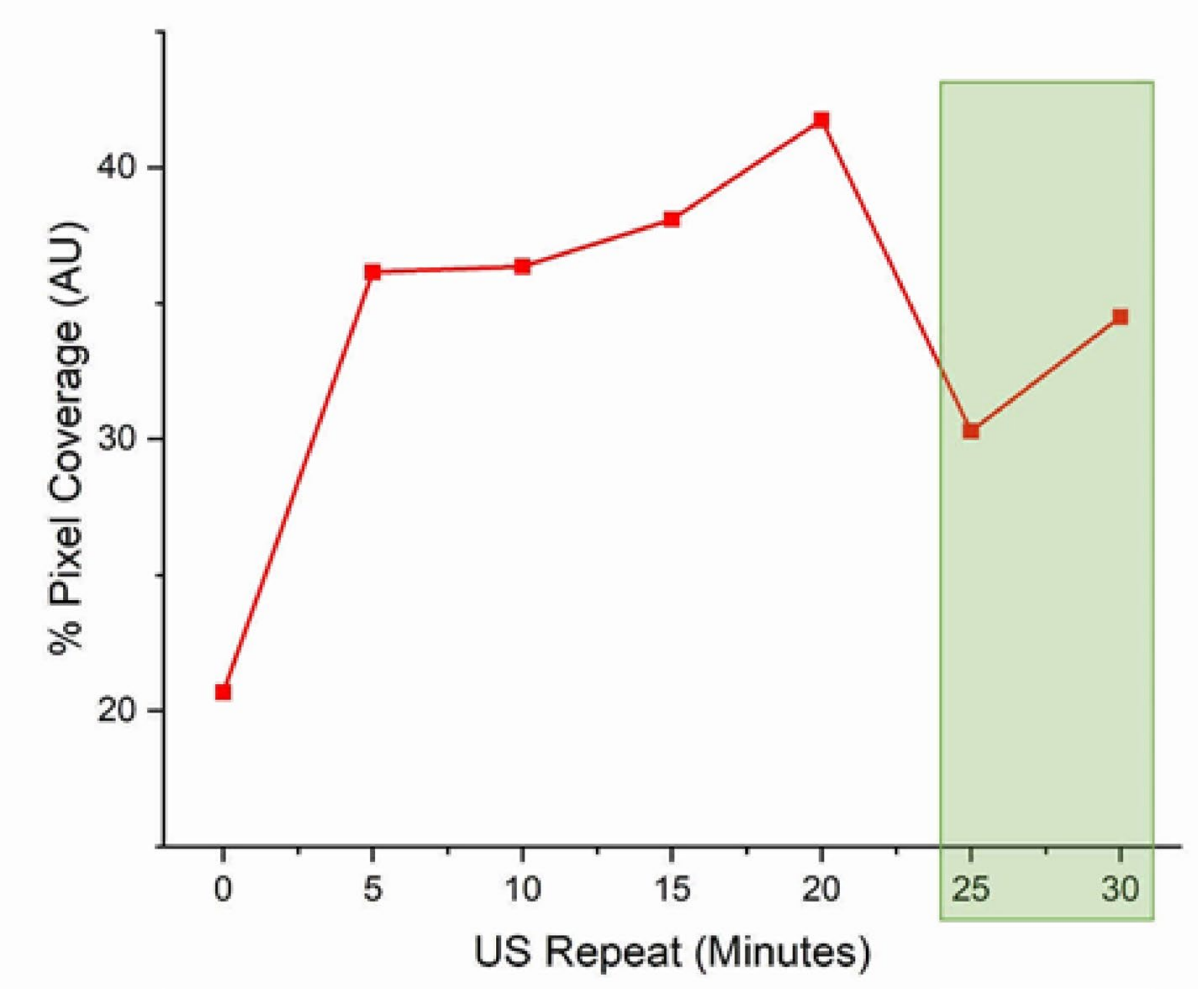
Data analysed from fluorescence images show % bright pixel area coverage in the PDAC cultures exposed to repeated US treatment at 5-min intervals for 30 min. The green shaded box indicates the time when microbubbles likely depleted from the culture reservoirs, and no subsequent influx of microbubbles into the culture from the media channels.

Clinically, US has been applied repeatedly after administering gemcitabine and microbubbles to increase the therapeutic delivery of gemcitabine, thereby reducing tumour size and growth and prolonging the quality of life of PDAC patients.^40,74^ First, we investigated the effect of the penetration of microbubbles into the PDAC culture as a function of repeated US exposure. Continued matrix disruption might increase the amount of gemcitabine flowing into the PDAC culture and, therefore, enhance the therapeutic effect. PDAC cells were seeded into the 5-channel device, cultured for 21 days, and treated with DPPC: DSPE-PEG2000: ATTO 488 microbubbles diluted in a 1:10 ratio with DMEM/10% FBS. After perfusing MBs into the device, US was applied every 5 min for 30 min, and fluorescence images were taken at each time point. Figures 5 and 6 show the fluorescence images for each time point and the percentage area coverage of bright pixels from fluorescence images of the PDAC cultures with increased US exposure, from 0 to 30 min, at 5-min intervals. Figure 6 shows an increase in fluorescence signal detected in the PDAC culture between the 1st US exposure (0 time point; ~ 20% coverage) and the 2nd US exposure after the first 5-min incubation (~ 35%), through to the 30-min duration for the repeated US exposure treatment. Figure 3B shows a representative image of the PDAC culture just before US exposure. The increase in fluorescence signal becomes more gradual between the 2nd and 4th US exposure (between 5 and 15 min of US repeat; maximum ~ 40%) before declining off between the 5th and 6th US Exposure (~ 28–32%), at time points of 25 and 30 min, respectively. The increase in fluorescence signal provides indirect evidence of matrix disruption, indicating microbubble cavitation and improved distribution and interstitial flow of microbubbles into the culture model. The bursting of the bubbles likely disrupts the PDAC matrix, allowing interstitial flow to replenish fluorescent bubbles within the matrix spaces. As the US is repeated, the effect increases with more matrix disruption, resulting in increased interstitial flow and matrix space and interactions with the cells. At the 5^th^ exposure, at a time point of 20 min, the fluorescence intensity appears to fall to around 30% coverage. If this were due to a plateau in the matrix disruption, one would expect the fluorescence intensity to also plateau. This effect is likely due to microbubbles in the media channels not being replaced by microbubbles in the reservoirs. Bubbles are inherently buoyant, and a 1—2 µm diameter bubble will take approximately 45 min to rise 1 cm^75^. Therefore, by the 25-min time point, many of the bubbles in the reservoirs will have risen away from the chip inlet and, consequently, will not replenish the microbubbles in the channels, thus creating a drop in the fluorescence signal.

**Fig. 7:**
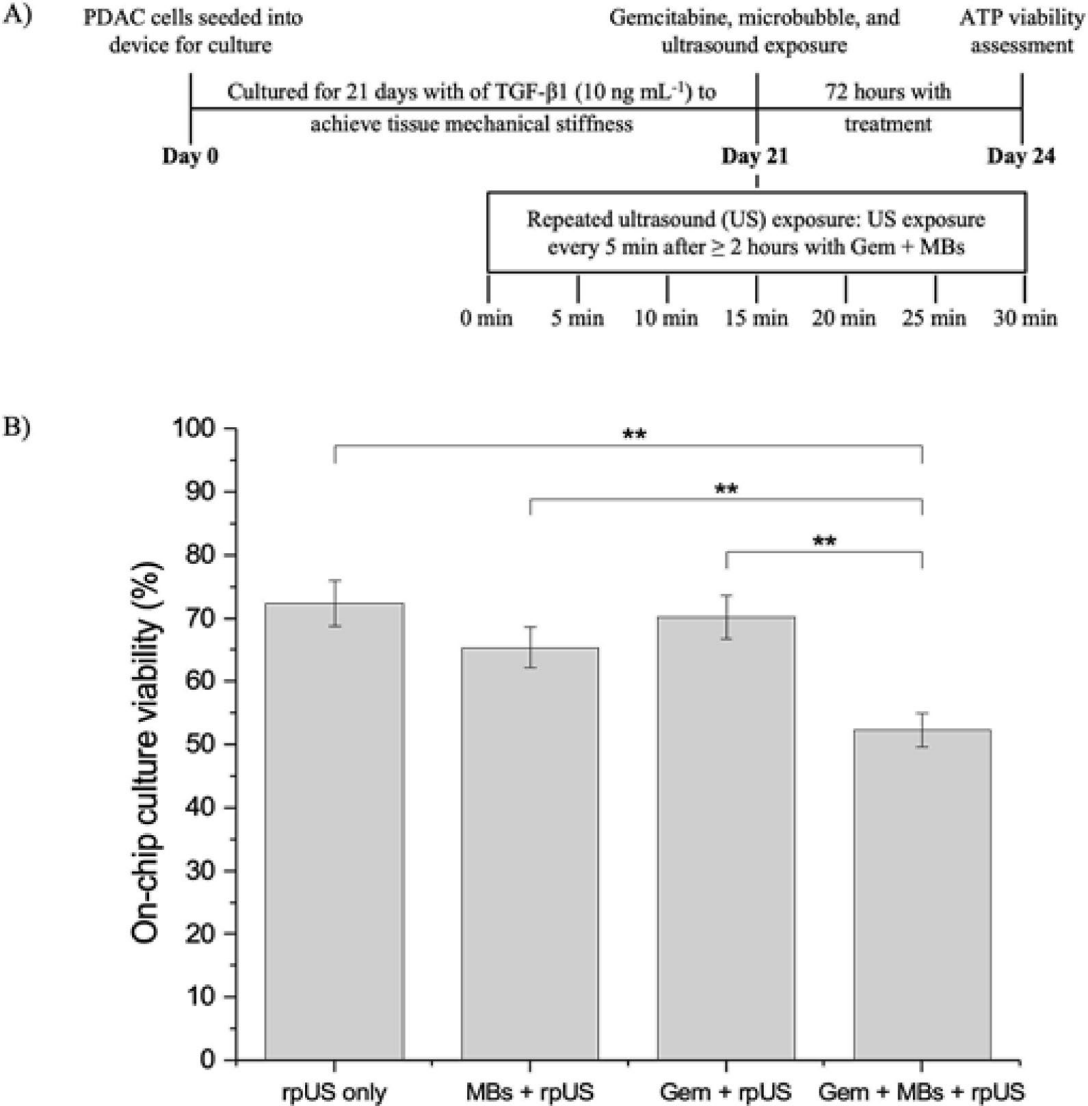
(**A**) Schematic of the treatment regime for the 21-day microfluidic PDAC culture with repeated US exposure. (**B**) ATP viability assessment of the PDAC culture model treated with 31.25 µM gemcitabine, microbubbles, and repeated ultrasound exposure together. *N* = 3 microfluidic cultures for the repeated ultrasound exposure only (rpUS only), the microbubbles and repeated ultrasound exposure together (MBs + rpUS), and the gemcitabine and repeated ultrasound exposure together (Gem + rpUS) treatments, and *n* = 4 microfluidic cultures for the gemcitabine, microbubbles, and repeated ultrasound exposure together (Gem + MBs + US) treatment all generated from three separate seeding settings. ** *p* ≤ 0.01, one-way ANOVA followed by Tukey’s multiple comparison test.

Next, we investigated if this disruption of the matrix with repeated microbubble and US exposure translated to an increased effect in gemcitabine treatment of the PDAC cultures. Similar to patient studies by Dimcevski et al. and Kotopoulis et al. ^40,74^, our microfluidic PDAC models were treated with gemcitabine and microbubbles with repeated US for 30 min. PDAC cells were seeded into the 5-channel device, cultured for 21 days, and treated with DPPC: DSPE-PEG2000: ATTO 488 microbubbles diluted in a 1:10 ratio with DMEM/10% FBS/31.25 µM gemcitabine solution. A US exposure regime identical to the one employed for disruption assessment was used. ATP culture viability was assessed after a 72-h incubation. Figure 7 shows the percentage viability of the cultures with gemcitabine, microbubbles, and repeated US exposure together (Gem + MBs + rpUS) compared to controls, repeated US exposure only (rpUS only), microbubbles and repeated US exposure together (MBs + rpUS), and gemcitabine and repeated US exposure together (Gem + rpUS). All data were normalised to positive and negative controls. rpUS only showed a 28% decrease in PDAC viability compared to controls, indicating that repeated US exposure can have its own therapeutic effect. The addition of MBs to rpUS (MBs + rpUS) had an additional 7% decrease in PDAC viability (35% decrease in PDAC viability compared to controls). This is likely due to the effect of microbubbles close to cells, as shown in Supplementary Fig.4(which shows still images from Supplementary Video 1, which displays microbubbles flowing into.

into the culture chamber with our model from the medium channel, present, and therefore close to cells). This causes membrane disruption through inertial cavitation, as inertial cavitation is most effective when microbubbles are near the cells. For Gem + rpUS, this showed an additional decrease in PDAC viability of 2—3% compared to rpUS only, or 30% compared to controls, due to the action of Gem as a chemotherapeutic. However, the full combination of rpUS, microbubbles and Gem (Gem + MBs + rpUS) showed an additional decrease in PDAC viability of 13% compared to MBs + rpUS, or a total decrease in the viability of 48% compared to non-treated 21-day PDAC culture. If we revisit the results from a single US exposure **(**Fig. 4A**)**, the single exposure of US with microbubbles and Gem (Gem + MBs + US) resulted in a 39% reduction in PDAC viability. Thus, the rpUS exposure under the same conditions resulted in an additional 9% reduction in PDAC viability. These results demonstrate the positive therapeutic effect of microbubbles co-delivered with the drug, following repeated US exposure, compared to the drug alone or other combinations of microbubbles, US, and drugs. Microbubble-mediated drug delivery, as demonstrated by this organ-on-a-chip device, shows promise as a drug delivery route to disrupt the dense, fibrotic stroma of PDAC and enhance therapeutic efficacy.

The differences in viability for all treatments between 2D cultures and the 3D 21-day PDAC culture grown in the microfluidic device highlight the importance of the dense, rigid stroma in tumours resistance to therapy and how this needs to be reflected in the models used for drug assessment. We have also demonstrated that microbubbles and US can be used to disrupt the PDAC matrix and increase the interstitial flow into the model. While a single treatment of microbubbles and US did not have a significant impact on the effectiveness of gemcitabine, repeated exposure over 30 min did significantly increase gemcitabine effect and reduce PDAC culture viability, which is in line with results observed in patient studies.

## Conclusion

This paper aimed to investigate the use of microbubbles and US to increase the effects of gemcitabine in our microfluidic PDAC culture model. Our 5-channel microfluidic PDAC culture model recapitulates the PDAC tumour mechanics with a rigid, collagenous tumour microenvironment and reduced interstitial flow. This made it an improved model for investigating increased gemcitabine efficacy with microbubbles and US. PDAC cultures presented in the literature demonstrate how cancer-stroma interactions influence therapeutic resistance, without considering the critical biophysical hallmarks that contribute to the ineffectiveness of chemotherapeutics. Owing to their sonoporation effects, creating transient pores in cell membranes, the use of microbubbles with US exposure has been shown to enhance drug uptake and effects in vitro and in vivo. Here, microbubbles exposed to repeated US were used to enhance the interstitial delivery of gemcitabine, and their effect on the microfluidic PDAC culture model, a treatment investigated in patient studies. Repeated US exposure with microbubbles and gemcitabine caused a 48% reduction in PDAC viability compared to a 39% reduction for the single exposure. Future experiments would investigate both a range of gemcitabine concentrations, identify optimal US and sonoporation parameters for maximum disruption to the PDAC matrix and perform immunostaining for stromal components such as collagen type I to confirm a disruption of the culture stroma. Taken together, this study highlights the importance of modelling the biophysical microenvironment of the PDAC tumour for the successful testing of novel drug delivery mechanisms to improve and better predict patient outcomes.

## Supplementary Information

Below is the link to the electronic supplementary material.

Supplementary Material 1

## Data Availability

The raw data associated with this article can be found available at 10.5518/1218.
