# Supplementary material for: Effect of microbubble-assisted gemcitabine delivery with repeated ultrasound exposure in a pancreatic cancer organ-on-a-chip model
Source: Sci Rep. 2025 Dec 12;15:44035. doi: 10.1038/s41598-025-30612-2 (PMC12715219; doi:10.1038/s41598-025-30612-2)
Supplement: Supplementary file 1 — Supplementary Material 1 [file 41598_2025_30612_MOESM1_ESM.docx]

**Supplementary Information**

Effect of microbubble-assisted gemcitabine delivery with repeated ultrasound exposure in a pancreatic cancer organ-on-a-chip model

Delanyo Kpeglo, Malcolm Haddrick, Margaret A. Knowles, Stephen D. Evans, and Sally A. Peyman*

* Correspondence to Dr. S. A. Peyman, [S.Peyman@hw.ac.uk](mailto:S.Peyman@hw.ac.uk)

**Supplementary Videos**

**Supplementary video 1**. The flow of microbubbles between the micropillars of the 5-channel microfluidic device into the culture chamber with the PDAC culture.

**Supplementary video 2**. The flow of microbubbles between the micropillars of the 5-channel microfluidic device into the culture chamber with 6 – 9 mg mL^-1^ BME gel only.


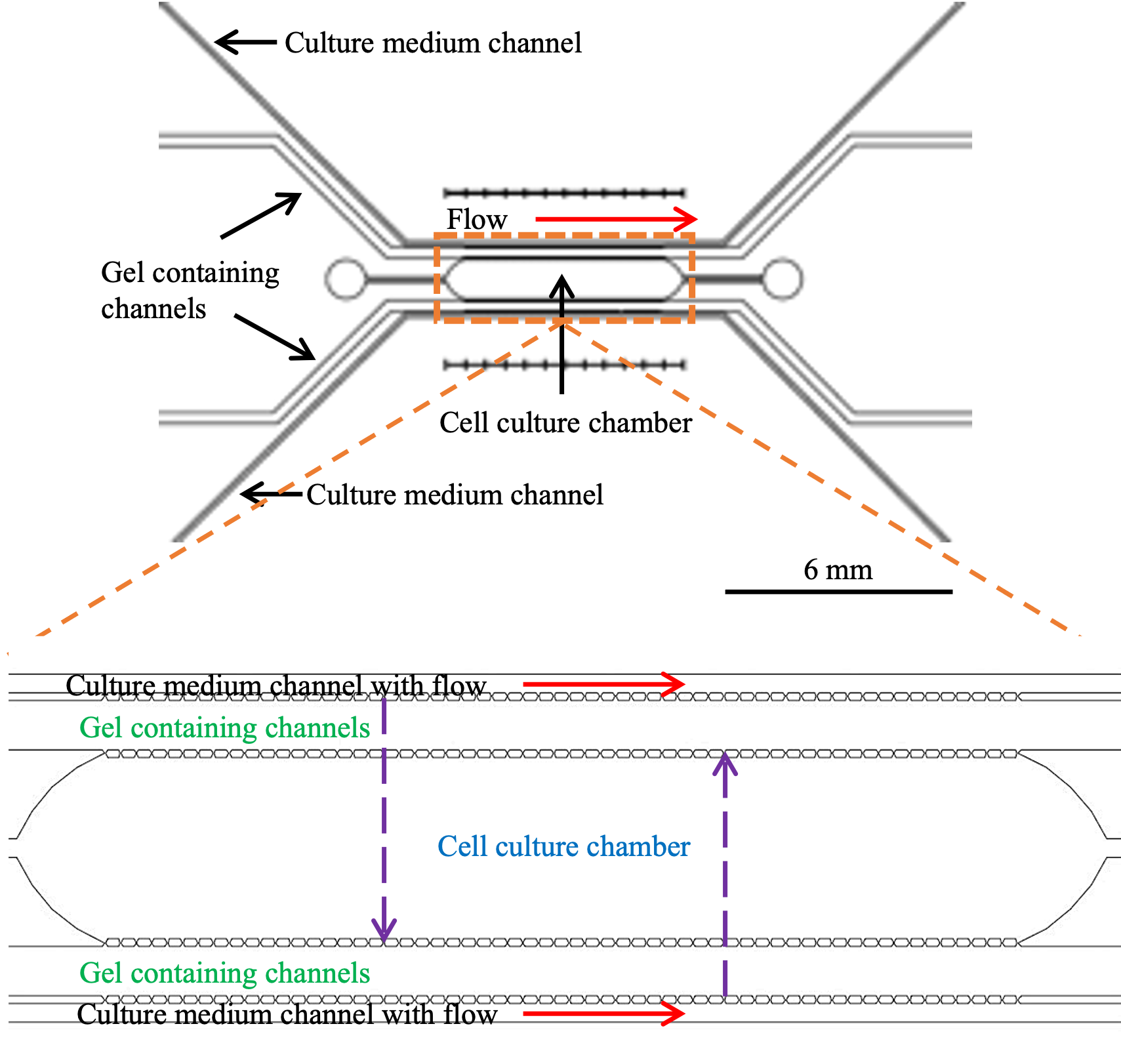


**Supplementary Figure 1**. The 5-channel microfluidic device for the pancreatic ductal adenocarcinoma (PDAC) culture model. The 100 μm high device is composed of a 1 mm × 6 mm (w × l) central culture chamber, two 275 µm wide gel containing channels (to achieve long-term cell culture over a 21-day period), and two 100 µm wide culture media channels. Micropillars with a 5 µm interspace were included at the boundary of the channels to help confine contents in the respective channel and for the stable diffusion of culture medium into the culture chamber. The presence of the micropillars also permitted the stable diffusion of medium (represented by the purple arrows) into the culture chamber for PDAC culture.


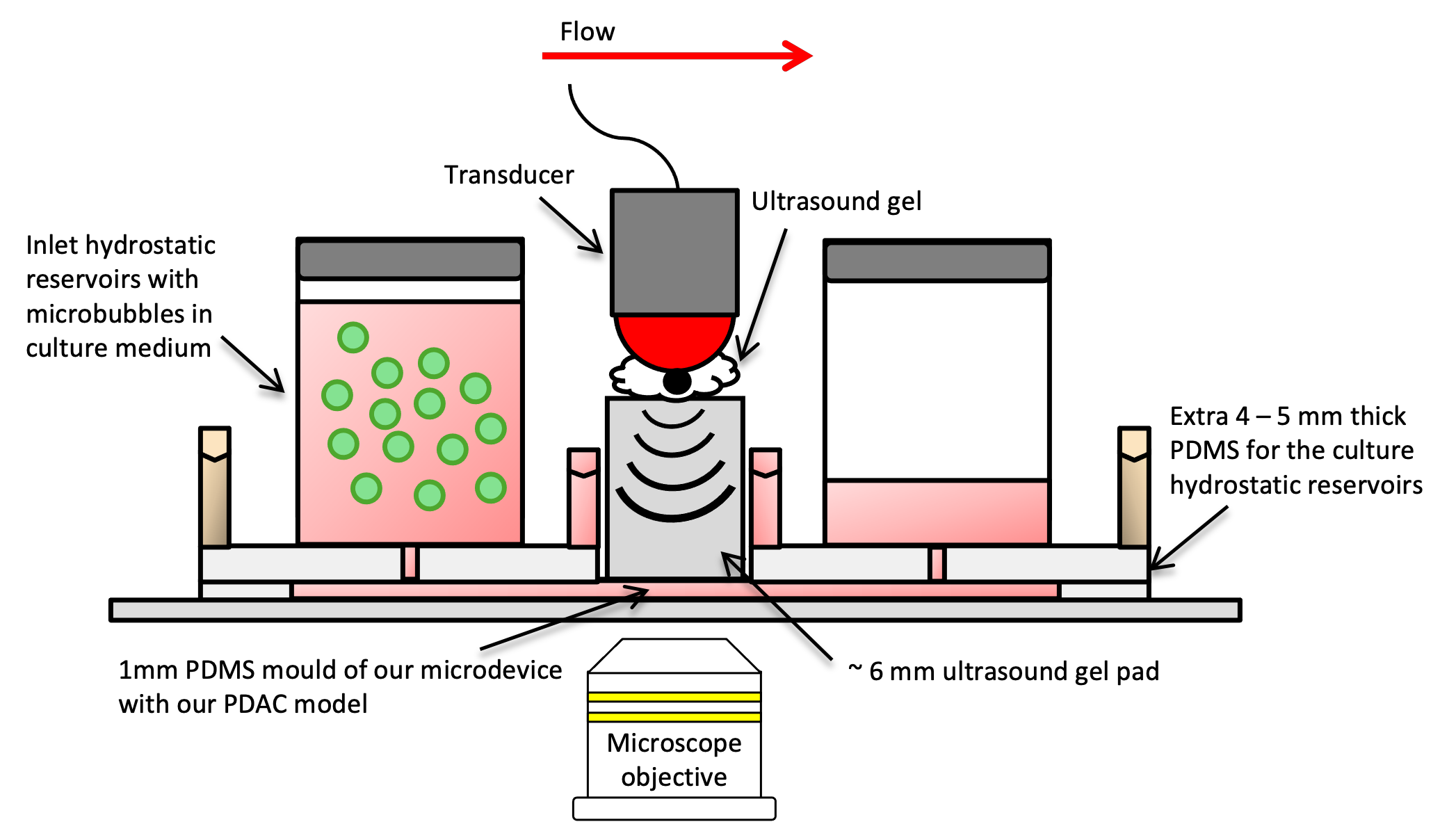


**Supplementary Figure 2**. Schematic of the ultrasound setup showing a 1 mm thick PDMS layer of the microdevice with the PDAC culture model, and a gel pad about 6 mm thick on the culture area for ultrasound coupling and application.



**Supplementary Figure 3**. Bright field images of the PDAC culture and BME only gel, before and after US exposure without microbubbles, showing no significant disruption of the gel or the cultures. Scale bar, 200 µm.





**Supplementary Figure 4**. Still images from Supplementary video 1, which shows the flow of microbubbles into the PDAC culture model (orange arrow) from the culture medium channel (red arrow) through the interspace of the 5 µm micropillars. The images show some of the microbubbles (indicated by the white arrow) surrounding the cells in our device, with areas where microbubbles were observed to be concentrated. Scale bar, 20 µm.
